# Stress entropic load: New stress measurement method?

**DOI:** 10.1371/journal.pone.0205812

**Published:** 2018-10-18

**Authors:** Filip Zlámal, Peter Lenart, Daniela Kuruczová, Tomáš Kalina, Gabriel de la Torre, Miguel A. Ramallo, Julie Bienertová-Vašků

**Affiliations:** 1 Research Center for Toxic Compounds in the Environment (RECETOX), Faculty of science, Masaryk University, Brno, Czech Republic; 2 Department of Pathological Physiology, Faculty of Medicine, Masaryk University, Brno, Czech Republic; 3 Department of Athletics, Swimming and Outdoor Sports, Faculty of Sports Studies, Masaryk University, Brno, Czech Republic; 4 Department of Psychology, University of Cadiz, Campus Rio San Pedro, Puerto Real, Cadiz, Spain; US Army Research Institute of Environmental Medicine, UNITED STATES

## Abstract

While stress is a widely utilized concept, no direct methods facilitating its measurement are currently available. In our previous work we proposed stress entropic load (SEL) as a potential new marker of stress response in the human body. However, at that time no method for SEL measurement existed. In this pilot study we devised and then tested methodology for SEL measurement. Healthy male participants were monitored by indirect calorimetry and thermography while resting and subsequently while under prolonged mental effort. The acquired data was then used to calculate the temporal development of SEL for each participant. Our results show that SEL production increased significantly in participants subjected to prolonged mental effort. Furthermore, we observed that the calculated development of SEL over time may be used to accurately determine the time point at which participants started performing stressful tasks.

## Introduction

Chronic environmental influences undoubtedly play an important role in shaping human health. However, while many explanations have been proposed, no completely satisfactory theory describing the interaction between organisms and the surrounding environment is currently available.

One of the most robust existing explanations focusing on the effect of chronic environmental influences is the stress theory developed by Hans Selye. In 1936 Selye noted that when organisms are severely damaged a typical three-stage syndrome appears regardless of the cause of the damage (surgery, chemical, etc.) [1]. Although this article did define the concept of the general adaptation syndrome, the paper did not mention the concept of “stress”–nor did it provide a useful tool for its measurement. Ten years later, Selye published a full account of his further experimental findings entitled “The general adaptation syndrome and diseases of adaptation” [2] where he defined stress as the “nonspecific response of the body to any demand made on it”. Within this paper Selye’s concept of stress „denotes”the bodily reaction to environmental stimuli. The primary weakness of Selye’s concept of stress is that it ignores interindividual differences in the perception of stressor or the effector pathway of the general adaptation to this stressor. Indeed, dissatisfaction with the vagueness of his definition of what constitutes stress plagued Hans Selye throughout his entire scientific career. On the other hand, this broad generalized view might be considered a major strength of his theory, especially as it corresponds rather well with our current understanding of interactions of an individual with his/her environment. However, any direct whole-body measurement of stress response remains elusive and available methods facilitate only an indirect measurement of stress based on parameters presumably associated with stress response severity such as blood pressure.

The concept of allostatic load provides a rough estimation of stress adaptation costs, in that it reflects the wear and tear experienced by individuals coping with repeated stressors and perturbations in a given system caused by environmental influences [3]. This concept of stress measurement, proposed by McEwen and Wingfield, facilitates the approximation of stress response using indirect parameters associated with HPA axis activation. While substantial limitations exist due to the indirect character of measured values, the methods based on allostatic load or its components measurements represent currently the most robust methodology for measuring biological correlates of stress.

Using entropy as a departure point, we previously proposed a holistic thermodynamic model of health and disease whose universal character incidentally corresponds to Selye’s general theory of stress but which does not contradict currently known concepts of environmental interactions with an individual or a population [4]. The proposed model enables the calculation of stress as well as the quantification of stress over a given period. The calculation of stress in this model is based on the cumulative production of entropy associated with a given stressor or a combination of stressors in a specific individual and/or in a specific regulatory feedback loop in a given interval.

In our previous study [4] we introduced stress entropic load (SEL), a variable designed to facilitate the objective physical measurement of the stress load of a human body. Mathematically, SEL is an explicitly time-dependent function expressed implicitly by different types of heat and temperature changes. Furthermore, in our previous article, we also described an equation calculating approximate SEL for a short measurement interval [4]. In this article we present a novel approach to measure SEL development in humans and we further test this approach on healthy male volunteers subjected to prolonged mental effort.

## Methods

### Derivation of SEL variables

The variables necessary for calculating SEL are typically time-dependent. However, these variables may also be calculated from other measurable variables based on known formulas. Specifically, heat exchanges can be expressed as follows, with all variables appearing in Eqs 2–7 listed in Table 1.

1. Radiation heat exchange rate
  $${\dot{Q}}_{R}(t)={\dot{Q}}_{R,in}(t)-{\dot{Q}}_{R,out}(t)=\varepsilon A_{e}\sigma_{SB}T_{s}{}^{4}-\eta A_{e}\sigma_{SB}T_{A}{}^{4}$$ (1)
2. Convection heat loss rate
  $${\dot{Q}}_{CNV}(t)=1.87A_{e}\sqrt{\frac{p}{p_{0}}}{\left(T_{S}-T_{A}\right)}^{\frac{5}{4}}$$ (2)
3. Evaporation heat loss rate
  $${\dot{Q}}_{E}(t)={\dot{Q}}_{D}(t)+{\dot{Q}}_{SW}(t)$$ (3)
  - by diffusion
    $${\dot{Q}}_{D}(t)=A_{e}\lambda \mu (p_{S}-p_{A})=A_{e}\lambda \mu \left[p(T_{S})-H_{R}p(T_{A})\right],$$ (4)
    with $p(T)=611.21\text{exp}\left[\left(18.678-\frac{T-273.15}{234.5}\right)\frac{T-273.15}{T-16.01}\right]$
  - by sweating
    $${\dot{Q}}_{SW}(t)=\left[\left(w_{in}-w_{f}\right)-\left(f+u\right)-0.019\langle M\left(O_{2}\right)\rangle \frac{5866-p_{A}}{133.322}t\right]\frac{\lambda }{60t}$$ (5)
4. Respiration heat loss rate
  $${\dot{Q}}_{RES}(t)=A_{e}\left[0.0014M\left(T_{RES}-T_{A}\right)+0.0017M\frac{7826-p_{A}}{133.322}\right]$$ (6)

**Table 1:** Quantities appearing in formulas 1 to 6.

Variable	Unit	Description
A_e_	m^2^	effective skin area
T_S_	K	average skin temperature
T_RES_	K	respirated heat temperature
T_A_	K	ambient air temperature
Tb	K	body temperature
Tc	K	core body temperature
Ts	K	skin temperature
p	Pa	atmospheric ambient air pressure
p_S_	Pa	saturated air vapor pressure at skin temperature
p_A_	Pa	saturated air vapor pressure at ambient air temperature
H_R_	-	relative humidity
w_in_	g	initial weight
w_f_	g	final weight
f	g	fluid/food intake
u	g	urine/feces loss
〈*M* (*O*_2_)〉	l·min^-1^	average oxygen uptake rate during measurement
*Ṁ*(*O*_2_)	mol·s^-1^	O_2_ uptake rate
*Ṁ*(*CO*_2_)	mol·s^-1^	CO_2_ liberation rate
t	min	measurement duration
M	W	metabolic heat production
ε	-	skin emissivity, ε = 0.98
η	-	skin absorptivity, η = 0.98
σ_SB_	-	Stefan-Boltzmann constant, σ_SB_ = 5.670367 · 10^8^ Wm^−2^K^−4^
p_0_	-	atmospheric pressure at standard conditions, *p*_0_ = 101325*Pa*
λ	-	latent heat of evaporation sweat, λ = 2430*Jg*^−1^
μ	-	skin permeance coefficient, μ = 1.270608·10^−6^ *gs*^−1^*m*^−2^*Pa*^−1^
$\sigma_{O_{2}}$	-	oxygen entropy content, σ_O2_ = 9.14785Jl^−1^ *K*^−1^
$\sigma_{CO_{2}}$	-	carbon dioxide entropy content,$\sigma_{CO_{2}}=9.53868Jl^{-1}K^{-1}$

All variables used in Eqs 2–7 are listed in Table 1.

Including the above equations, i.e. 1 to 6, to the previously published formula [4] provides the following derived formula for the calculation of SEL:

$$\begin{array}{r} \Delta s_{SEL}(t)=\frac{1}{w}\int_{0}^{t}\{\frac{M(s)}{T_{B}(s)}\left(1-\frac{{\dot{T}}_{B}(s)}{T_{B}(s)}\right)+\varepsilon A_{e}\sigma_{SB}T_{s}{\left(s\right)}^{4}\left(\frac{1}{T_{S}(s)}-\frac{1}{T_{B}(s)}\right)-\eta A_{e}\sigma_{SB}T_{A}{\left(s\right)}^{4}\left(\frac{1}{T_{A}(s)}-\frac{1}{T_{B}(s)}\right)+\\ +1.87A_{e}\sqrt{\frac{p}{p_{0}}}{\left(T_{S}\left(s\right)-T_{A}(s)\right)}^{\frac{5}{4}}\left(\frac{1}{T_{S}(s)}-\frac{1}{T_{B}(s)}\right)+\\ +A_{e}\lambda \mu [611.21\;\text{exp}\left(\left(18.678-\frac{T_{S}(s)-273.15}{234.5}\right)\frac{T_{S}(s)-273.15}{T_{S}(s)-16.01}\right)-\\ -H_{R}611.21\;\text{exp}\left(\left(18.678-\frac{T_{S}(s)-273.15}{234.5}\right)\frac{T_{A}(s)-273.15}{T_{A}(s)-16.01}\right)]\left(\frac{1}{T_{C}(s)}-\frac{1}{T_{B}(s)}\right)+\\ +A_{e}M\left(s\right)[0.0014\left(T_{RES}(s)-T_{A}(s)\right)+0.0017(58.7-\\ -\frac{1}{133.332}H_{R}611.21\;\text{exp}\left(\left(18.678-\frac{T_{A}(s)-273.15}{234.5}\right)\frac{T_{A}(s)-273.15}{T_{A}(s)-16.01}\right))]\left(\frac{1}{T_{RES}(s)}-\frac{1}{T_{B}(s)}\right)+\\ +\frac{{\dot{T}}_{B}(s)}{T_{B}{\left(s\right)}^{2}}\int_{0}^{s}\left(\varepsilon A_{e}\sigma_{SB}T_{s}{\left(s\right)}^{4}-\eta A_{e}\sigma_{SB}T_{A}{\left(s\right)}^{4}\right)dr+\\ +\left(\frac{{\dot{T}}_{B}(s)}{T_{B}{\left(s\right)}^{2}}-\frac{{\dot{T}}_{S}(s)}{T_{S}{\left(s\right)}^{2}}\right)1.87A_{e}\sqrt{\frac{p}{p_{0}}}\int_{0}^{s}{\left(T_{S}\left(r\right)-T_{A}(r)\right)}^{\frac{5}{4}}dr+\\ +\left(\frac{{\dot{T}}_{B}(s)}{T_{B}{\left(s\right)}^{2}}-\frac{{\dot{T}}_{C}(s)}{T_{C}{\left(s\right)}^{2}}\right)A_{e}\lambda \mu \int_{0}^{s}[611.21\;\text{exp}\left(\left(18.678-\frac{T_{S}(r)-273.15}{234.5}\right)\frac{T_{S}(r)-273.15}{T_{S}(r)-16.01}\right)-\\ -H_{R}611.21\;\text{exp}\left(\left(18.678-\frac{T_{A}(r)-273.15}{234.5}\right)\frac{T_{A}(r)-273.15}{T_{A}(r)-16.01}\right)]dr+\\ +\left(\frac{{\dot{T}}_{B}(s)}{T_{B}{\left(s\right)}^{2}}-\frac{{\dot{T}}_{RES}(s)}{T_{RES}{\left(s\right)}^{2}}\right)A_{e}\int_{0}^{s}M\left(r\right)[0.0014\left(T_{RES}(r)-T_{A}(r)\right)+0.0017(58.7-\\ -\frac{1}{133.332}H_{R}611.21\;\text{exp}\left(\left(18.678-\frac{T_{A}(r)-273.15}{234.5}\right)\frac{T_{A}(r)-273.15}{T_{A}(r)-16.01}\right))]dr-\\ -\sigma_{{\text{O}}_{2}}\dot{\text{M}}\left({\text{O}}_{2}\right)\left(s\right)+\sigma_{\text{C}{\text{O}}_{2}}\dot{\text{M}}\left(\text{C}{\text{O}}_{2}\right)\left(s\right)\}ds-\sigma_{prod,no\;stress}t \end{array}$$ (7)

This derived SEL relation comprises three kinds of variables:

1. time-dependent: T_A_, T_B_, T_C_, T_S_, M, Ṁ(O_2_), Ṁ(CO_2_), H_R_
2. constants: ε, η, σ_SB_, p_0_, λ, μ
3. time-dependent quantities which may be considered constant under certain conditions: A_e_, w, T_RES_, p, $\sigma_{O_{2}}$, $\sigma_{{CO}_{2}}$, *σ*_*prod*,*no stress*_

The formula 7 enables us to calculate SEL from measurements of temperatures of a body, ambient air characteristics and breathing air exchange rates. This can be realized using indirect calorimetry methods (for measuring metabolic activity M, oxygen uptake and carbon dioxide liberation rates Ṁ(O_2_) and Ṁ(CO_2_)) and thermography (measuring temperatures of a body T_B_, T_C_ and T_S_). Characteristics of an ambient air T_A_ and H_R_ can be obtained by a special thermometer as well as atmospheric pressure p is measurable by a barometer.

### Experimental validation of SEL as a marker of prolonged mental effort

#### Subjects

We recruited 12 healthy male participants aged 20–30 years. The first 10 participants were part of the group exposed to the prolonged mental effort induced by WinSCAT tool (see below). The participants 11–12 were part of a control group which underwent the measurement without doing tasks requiring mental effort. The study was conducted at the Faculty of Sports Studies of Masaryk University in Brno, Czech Republic. Informed consent was obtained from all patients prior to the beginning of the experiment and the study was approved by the Ethics Committee of the Faculty of Sports Studies of Masaryk University. The characteristics of all study participants are summarized in Table 2.

**Table 2:** Basic anthropometric characteristics of study participants.

Participant No.	Age [y]	Weight [kg]	Height [cm]
1	20	88.7	183
2	23	71.2	175
3	27	86.2	176
4	30	88.6	176
5	28	85.5	189
6	20	80.9	172
7	21	65.5	174
8	22	69.4	176
9	25	89.3	186
10	21	75.7	185
mean ± SD	23.7 ± 3.6	80.1 ± 9.0	179.2 ± 5.9
11	26	78.3	178
12	27	66.0	180
mean ± SD	26.5 ± 0.7	72.2 ± 8.7	179.0 ± 0.4

#### Experiment—WinSCAT software

In order to induce prolonged mental effort, we used Spaceflight Cognitive Assessment Tool for Windows (WinSCAT). WinSCAT was developed as a tool designed to support medical operations at NASA’s Johnson Space Center, specifically to monitor the neurocognitive status of space crews [5–7]. The test is composed of several cognitive subtests which measure sustained concentration, working memory, attention, short-term memory, spatial processing, mathematical skills and processing efficiency. The current version of WinSCAT includes four cognitive subtests:

Mathematical Processing (MTH). The participant is required to solve a sequence of mathematical problems calling for addition and subtraction and to press either the right or left mouse button to determine if the correct response is greater or smaller than 5. The problems appear one at a time on screen, and each problem requires either addition or subtraction. Two scores quantify the performance of the participant. The mathematical processing response time which quantifies reaction speed and the mathematical processing accuracy which asses percentual accuracy of answers.

Running Memory Continuous Performance Test (CPT). During this test, single numbers are shown on the screen. Participants need to determine if the number shown is the same than the one immediately before it. Buttons of the mouse need to be pressed each time a number appears on screen except for the first one. Left or right clicks will be used to determine if the current number shown was shown before. Three scores quantify the performance of the participant. Repeating numbers response time which quantifies reaction speed, the repeating numbers accuracy which asses percentual accuracy of answers and the repeating numbers time misses which counts how many times participants didn’t answer.

Delayed Matching to Sample (MSP). In this test, first, a sample stimulus in the form of a large box with colored squares is shown on the screen. After a while, the sample box disappears and two comparison boxes appear side by side. Participants need to decide, which one of these two comparison boxes matches the sample box. Left or right mouse buttons need to be clicked. Two scores quantify the performance of the participant. The pattern memory response time which quantifies reaction speed and the pattern memory accuracy which asses percentual accuracy of answers.

Codes Substitution (CDS). In CDS test, a row of symbols and a row of numbers are shown on the screen. Each number has a symbol which appears in the box above the number. During the test a sample box with a symbol and a number will be displayed below the rows. For each trial, participants are asked to determine whether the sample box was shown with the correct corresponding symbol and to indicate their choice with a left- or right-click of the mouse. The CDS measures sustained attention and concentration, visual search, verbal learning, and recall. Two scores quantify the performance of the participant. The symbol memory response time which quantifies reaction speed and the symbol memory accuracy which asses percentual accuracy of answers.

All tests were designed for analysis using repeated measures. The interface is simplified so that all tests are presented in a for- mat allowing the user to respond by pushing either the left or the right mouse button [6].

#### Experiment—Preparation

Two days before the experiment, each participant was emailed a set of basic instructions, namely to: avoid alcohol consumption for at least 24 hours before the start of an experiment, avoid hard physical activity for at least 12 hours before the start of an experiment, get at least 8 hours of sleep and come on an empty stomach.

Participants were invited to arrive by 8.45 a.m. to the faculty examination room. Upon arrival, each participant read and signed the informed consent form. Subsequently, each participant from WinSCAT group was provided with a tablet installed with WinSCAT software; a basic demo version was used to demonstrate the utilized tests and to provide basic insight into the required procedure. Next, basic anthropometric characteristics (e.g. weight and height) were measured. In all experiments, the participants used WinSCAT software on a tablet with the connected computer mouse, to minimize physical effort and discomfort.

Afterward, the main part of an experiment started. Each participant was seated on a medical folding bed in a Semi-Fowler`s position for the entire duration of the measurement. Each participant was asked to remove clothing from the upper part of the body to facilitate the collection of thermograms by means of a thermal camera in order to record changes in body temperature (every 20 seconds). The participant’s legs were covered with thermoinsulating foil to eliminate heat loss from the lower part of the body. A silicon mask was attached to the participant’s face and connected to a spirometry system which continuously recorded breathing air exchange rates and metabolic activity. This setting provided the maximum possible comfort and minimized the physical activity of each participant throughout the experiment. During the entire duration of each experiment, ambient air characteristics were recorded intermittently every 15 minutes as well as the participant’s blood pressure and heart rate. The experiment started once the spirometry system and the thermal camera were turned on, i.e. approximately 30 minutes after the arrival of each participant. All devices utilized throughout the experiment are listed in Table 3.

**Table 3:** Technical devices and measured variables.

Device	Measured variable
spirometry system Cortex MetaLyzer 3B-R3	M, Ṁ(O_2_), Ṁ(CO_2_)
thermal camera FLIR SC640	T_C_, T_S_, T_B_
Inbody 720p	weight, height
thermometer KESTREL 4000	T_A_, H_R_
barometer GPB 2300	p
blood pressure monitor Hartmann Tensoval	blood pressure, heart rate

Average skin temperature T_S_ and average body temperature T_C_ were calculated from thermograms using FLIR Tools software, v. 6.3.17214.1005. The latter was estimated from the temperature of the supraclavicular fossa [8]. The temperature of the entire body was subsequently calculated using the following formula: T_B_ = 0.65T_C_+0.35T_S_ [9]. The temperature of respirated air T_RES_ was assumed to be constant and equal to 34°C throughout the experiment.

#### Experiment—Measurement

The measurement itself consisted of two parts. In the first part, the participant was asked to calm down and breathe freely to attain resting metabolic rate (RMR) defined by i) maximum deviation 10% of oxygen uptake rate, ii) maximum deviation 10% of carbon dioxide liberation rate, iii) maximum deviation 5% of respiratory quotient (RQ), continously during 5 minutes of measurement. In case a participant failed to satisfy all three criteria in the first 40 minutes of the measurement phase, the experiment continued as if RMR had been attained. The time interval containing information about RMR is called the RMR subphase. Data from this phase were then used to calculate the entropy production of a resting body as specified in Eq (7) as σ_prod, no stress_ as well as additional characteristics: average Ṁ(O_2_), average Ṁ(CO_2_), average M, average temperatures T_S_, T_C_, T_B_.

In the second part of the experiment, which started once RMR was attained or after 40 minutes of measurement, each participant from the WinSCAT group received a tablet with WinSCAT software and began to complete assigned tasks. The time limit for completing one series of tests (i.e., one run of the WinSCAT program) was 15 minutes; each participant was required to complete 12 series of tests. The completion of all tests thus took approximately 3 hours. The process may be divided into two subphases. The first subphase consists of the first 6 test series (i.e. the first 1.5 hours of the task) and serves as a learning part in which a participant acquires better control and insight. In the second subphase, consisting of the second batch of 6 test batteries (i.e. the second 1.5 hours of the task), the participant experiences stress due to the prolonged mental effort. This subphase is considered to be stressful for the purposes of this study. Accordingly, data obtained from this subphase were used to calculate entropy production during stressful experience.

#### SEL calculation

The development of SEL over time is calculated by subtracting the two time series. The first time series is extrapolated from data from the RMR subphase. Entropy production during the RMR subphase is extrapolated to the entire duration of the experiment and serves as a baseline entropy production value. The second time series contains data about entropy production from measurement during the entire course of the experiment.

Therefore, expected SEL production during the RMR subphase is zero, i.e.$\;{\left(\frac{dSEL}{dt}\right)}_{RMR\;subphase}\;=\;0$.

Calculating the slopes of SEL (i.e. average SEL change per unit of time) during the stressful subphase can lead to three scenarios:

1. ${\left(\frac{dSEL}{dt}\right)}_{stressful\;subphase}>0$: SEL increases with time; positive response in SEL production; SEL production is positive during the stressful subphase of the experiment; SEL increase is associated with stress induction,
2. ${\left(\frac{dSEL}{dt}\right)}_{stressful\;subphase}\;=\;0$: SEL remains constant; no response in SEL production; SEL production is zero during the stressful subphase of the experiment and SEL is thus not associated with stress induction,
3. ${\left(\frac{dSEL}{dt}\right)}_{stressful\;subphase}<0$: SEL decreases with time; negative response in SEL production; SEL production is negative during the stressful subphase of the experiment and SEL is thus negatively associated with stress induction.

#### Data analysis

For the purpose of the statistical comparison of selected characteristics between RMR and stressful subphases, average characteristics of obtained time series of Ṁ(O_2_), Ṁ(CO_2_), M, specific cumulative entropy production rate (cEPR) and SEL rate of the study participants were calculated for RMR and stressful subphases. The descriptive characteristics of the variables are expressed as mean ± standard deviation. Furthermore, we compared values of selected variables between RMR and stressful subphases using a paired t-test. Body temperatures T_S_, T_C_ and T_B_ were also calculated for the RMR subphase.

The calculation of the SEL time series required special treatment due to different time steps for different variables. The time series were first smoothed by means of kernel smoothing [10]. Then, all time series and other variables were imputed to the formula (7) and integrated by means of numerical integration using the rectangular rule [11]. Slopes in SEL time series were calculated using linear regression.

To test whether using WinSCAT software influenced the SEL development we constructed two mixed effects models. First model contained only time while second model contained time and group as independent variables (in both model SEL was considered as dependent variable, with "subject" as random effect). Consecutively, we tested the significance of the variable “group” by employing the principle of testing submodels via likelihood ratio test.

Data analysis was performed using statistical software R, v. 3.3.3. p-values less than 0.05 were considered statistically significant.

## Results

Data for all participants were obtained using the above mentioned procedure. The scores in WinSCAT (S1 Table) were assed for each participant. The variables measured during experimental procedures (S2 Table) were used to calculate the cEPR and SEL rate for RMR and stressful subphase respectively (Table 4). Our calculations show that all study participants exhibited higher cEPR and increased SEL rate during the stressful subphase in comparison with the RMR subphase.

**Table 4:** Comparison of cEPR and SEL rate between RMR and stressful subphases.

	RMR subphase	Stressful subphase	RMR subphase	Stressful subphase
Participant no.	cEPR [JK^-1^kg^-1^min^-1^]	cEPR [JK^-1^kg^-1^min^-1^]	SEL rate [JK^-1^kg^-1^min^-1^]	SEL rate [JK^-1^kg^-1^min^-1^]
1	0.136	0.225	0.005	0.489
2	0.134	0.198	-0.001	0.474
3	0.151	0.188	0.034	0.164
4	0.154	0.205	-0.006	0.168
5	0.175	0.206	0.000	0.091
6	0.169	0.220	-0.002	0.297
7	0.160	0.275	-0.002	0.754
8	0.160	0.198	0.001	0.170
9	0.144	0.191	-0.002	0.302
10	0.199	0.204	-0.009	0.042
mean ± SD	0.158 ± 0.019	0.211 ± 0.25	0.002 ± 0.012	0.295 ± 0.220
11	0.157	0.172	-0.002	0.021
12	0.202	0.227	-0.001	0.086
mean ± SD	0.179 ± 0.032	0.199 ± 0.039	-0.001 ± 0.000	0.053 ±0.046

In the next step, we statistically compared several selected variables between the RMR and the stressful subphase (Table 5). Several of the tested variables were significantly higher in the stressful subphase of the WinSCAT group: Ṁ(O_2_) (p < 0.001), Ṁ(CO_2_) (p < 0.001), M (p < 0.001), cEPR (p < 0.001) and SEL (p = 0.002). On the other hand, no significant differences in systolic and diastolic blood pressure or heart rate were established (p = 0.692; p = 0.800; p = 0.481 respectively). Interestingly, in the control group the Ṁ(O_2_) and Ṁ(CO_2_) were almost constant with time (S2 Table). Furthermore, we have found no statistically significant association between SEL change and and outputs of WinSCAT test for participants (S3 Table).

**Table 5:** Statistical comparison of selected variables between RMR and stressful subphases in WinSCAT group.

Variable	RMR phase	stressful subphase	mean difference*	95% CI for mean difference	p-value
Ṁ(O_2_) [l·min^-1^]	0.27 ± 0.03	0.33 ± 0.03	0.060	(0.04; 0.09)	**<0.001**
Ṁ(CO_2_) [l·min^-1^]	0.22 ± 0.04	0.28 ± 0.03	0.060	(0.04; 0.09)	**<0.001**
M [W]	90.2 ± 11.2	112.5 ± 11.0	22.30	(13.2; 31.4)	**<0.001**
cEPR [JK^-1^kg^-1^min^-1^]	0.158 ± 0.019	0.211 ± 0.025	0.053	(0.031; 0.075)	**<0.001**
SEL rate [JK^-1^kg^-1^min^-1^]	0.002 ± 0.012	0.295 ± 0.220	0.293	(0.135; 0.451)	**0.002**
BP systolic [mmHg]	124.4 ± 11.4	125.8 ± 6.3	1.400	(-6.4; 9.3)	**0.692**
BP diastolic [mmHg]	82.9 ± 9.6	83.4 ± 7.1	0.500	(-3.9; 4.9)	**0.800**
heart rate [min^-1^]	67.7 ± 7.7	66.5 ± 7.9	- 1.200	(-5.1; 2.6)	**0.481**

* Mean difference was calculated as stressful subphase—RMR subphase. Paired t-test was used in all instances.

**Fig 1:**
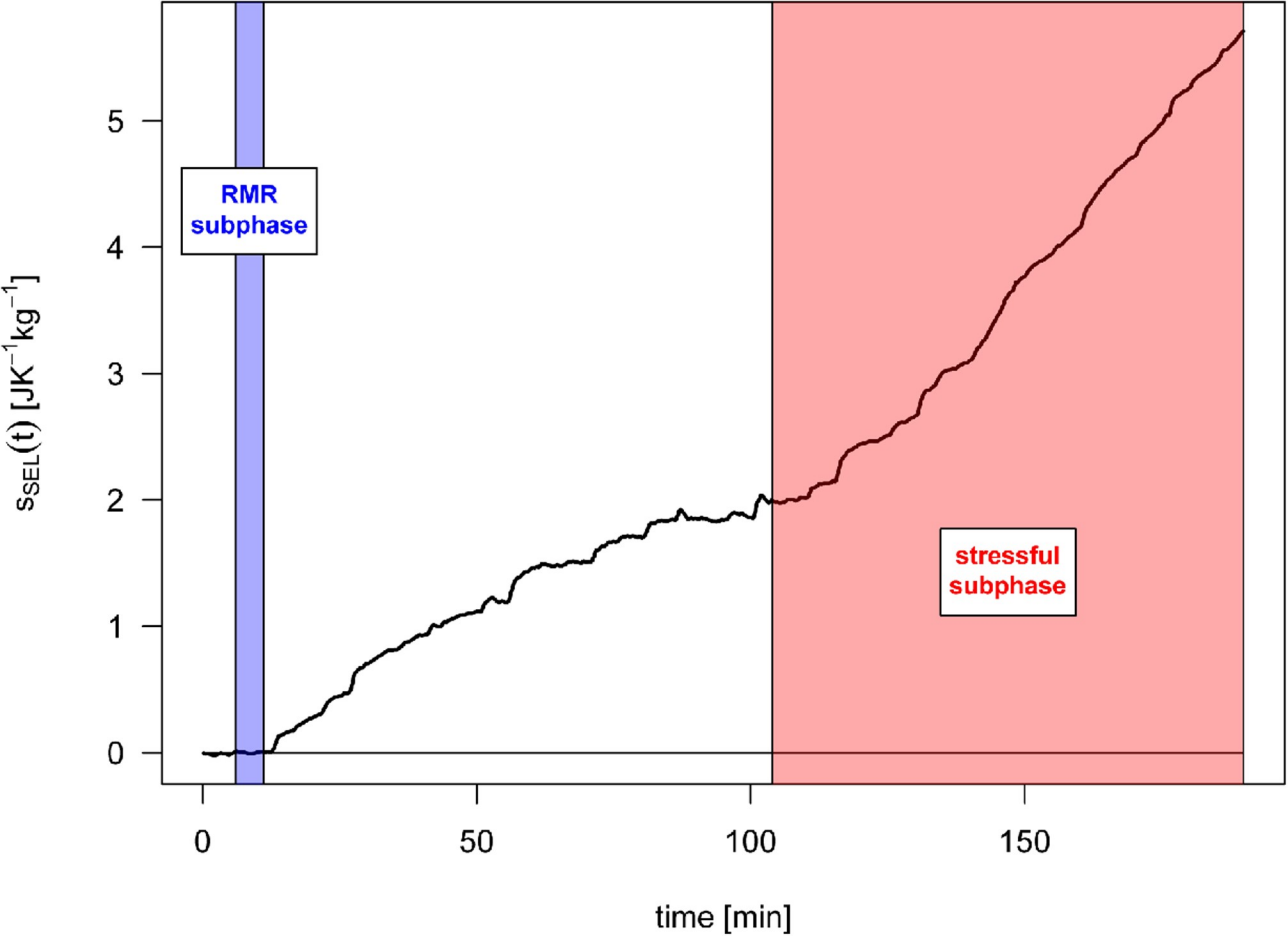
Example of calculated SEL time development of selected participant.

**Fig 2:**
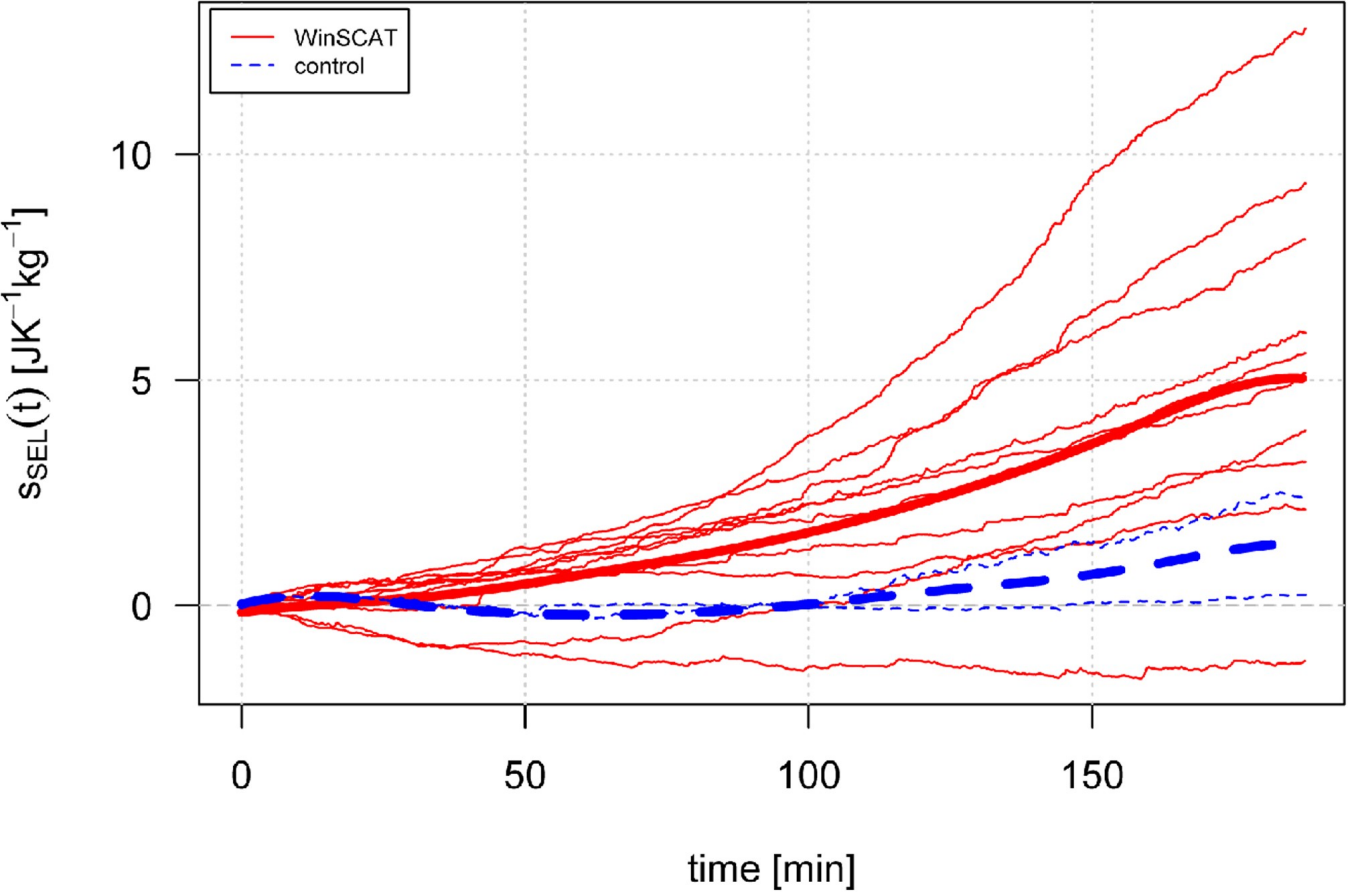
SEL development over time for all participants. Red lines represents a SEL development for participants in WinSCAT grop, while the blue lines represent SEL development for control group. Bold red and blue lines represent average SEL development for WinSCAT and control group respectively.

SEL has been observed to develop over time; calculated SEL development for one selected participant is shown in Fig 1. The temporal development of SEL has different dynamics for RMR and for the stressful subphases and can therefore be used on its own to determine the begnning of the stressful activity. The temporal development of SEL as an effect of the stressful phase has been observed similarly in all but one participant in the WinSCAT group (Fig 2).

To compare time development of SEL between WinSCAT and control group we constructed two mixed effects models. First model considered only effect of time while second model contained time and group as independent variables (in both model SEL was considered as dependent variable, with "subject" as random effect). Then we tested the significance for the “group” variable by the principle of testing submodels via likelihood ratio test. The two models were found to be highly significantly different (D = 5040.6, df = 9, p<0.001) meaning the group variable seems to be important explanatory variable for SEL variability.

## Discussion

In our previous study we hypothesized that specific entropy production and specifically the calculated SEL rate may function as stress marker. Consecutively, we have devised and tested method for measuring SEL rate. Our results further show that stress caused by prolonged mental effort induced by long and standardized repetitive usage of WinSCAT software under specific time constraints, significantly increases specific entropy production rate and more specifically, SEL rates. Furthermore, the temporal development of SEL over time can be used to accurately determine the time point at which each participant started performing stressful tasks. Our results thus support the notion that SEL can be used as a biology-based marker of prolonged mental effort. Interestingly, the prolonged mental effort caused by WinSCAT tasks does not increase systolic and diastolic blood pressure or heart rate, i.e., long-standing indirect stress markers. On the other hand, the group using the WinSCAT group had significantly higher Ṁ(O_2_) and Ṁ(CO_2_) while the control group showed only minimal difference in Ṁ(O_2_) and Ṁ(CO_2_) between the same time periods. Because, the respiratory volumes have been reported to increase with mental stress [12] this may be used to argue that the use of WinSCAT was more demanding for the participants. While our results support our hypothesis that SEL can be used as a stress marker they are not conclusive due to the limitations and pilot character of this study.

The primary strength of this study is that it provides a relatively easy method for the calculation of SEL by means of indirect calorimetry and thermography. Furthermore, our study is the first to explore the use of SEL as a marker of stress caused by prolonged mental effort in experimental fashion. However, we are aware that even though the results seem quite promising, our study suffers from several weaknesses. Arguably the most significant weakness of our study is that it employed a relatively small number of participants, all of whom were male. However, this study was intended only as a pilot study designed to test the method of measuring SEL by means of relatively inexpensive and easily accessible indirect calorimetry. The logical next step would thus be a more extensive study utilizing direct calorimetry. Nevertheless, our results constitute the first validation of the concept of SEL as a maker of stress caused by the prolonged mental effort which is an important precondition for any future investment into time-consuming and costly measurements utilizing direct calorimetry. We would also like to note that the absence of female participants in our study is the outcome of the fact that our experimental procedure required all participants to undergo the testing procedure with a naked torso.

While our data show that all participants had higher SEL rate during the stressful phase than during the RMR phase, in case of one participant SEL development over time was negative, as evident from Fig 2. The possible explanation could be this participant (number 10) did not attain RMR by satisfying all three criteria described in methods during first 40 minutes, and thus experiment continued as if RMR was attained, which could have led us to overestimate the basal entropy production for this participant.

Furthemore, while our results suggest a difference in SEL production between the WinSCAT and control group it is notable that in control group the SEL rate still increased with time. However, this may not be a limitation of SEL as a potential objective marker of stress. SEL was designed to measure all kinds of reactions to the environenmental conditions and siting with naked torso for four hours in Semi-Fowler`s possition while on spirometry with no task to keep you occupied for several hours may have consituted significant stress for the participants in our control group. Accordingly, even participants in control group reported that they precieved the experimental procedure as stresfull.

We believe that concept of SEL exhibits several important benefits in comparison with currently used markers of stress (e.g. parameters associated with the HPA axis). Most importantly, it measures stress in the whole organism instead of focusing on the response of selected organs. In addition, while many other stress markers can only be measured at specific time points, the development of SEL over time may be monitored continuously. Moreover, SEL provides an interesting opportunity to monitor stress in organisms without the HPA axis.

While our results suggest that SEL is measurable and serve as marker of stress induced by prolonged mental effort, this notion and feasibility of SEL as a general stress marker requires further testing. We believe that the concept of SEL could be applicable to a diverse set of situations where the monitoring of stress levels may be critical in terms of the prediction and prevention of an upcoming adverse events within the human body. For example, stress is a great challenge for manned spaceflight; according to Kanas & Manzey [13] astronauts encounter various types of stress, and an excessive amount of stress may have adverse effects on the health and well-being of astronauts. Thus, measurement of SEL may be useful for future long-duration human spaceflight including a manned mission to Mars. Others examples in which SEL measurement may be useful may include even military application, but we believe that SEL can be especially helpful and beneficial in medicine, e.g. as a method for the estimation of time to failure of ICU patients.

## Supporting information

S1 Table The WinSCAT results. (XLSX)

S2 Table The variables measured during experimental procedures. (XLSX)

S3 Table Relation between SEL changes and WinSCAT results. (XLSX)
